# Emergence of Microglia Bearing Senescence Markers During Paralysis Progression in a Rat Model of Inherited ALS

**DOI:** 10.3389/fnagi.2019.00042

**Published:** 2019-02-28

**Authors:** Emiliano Trias, Pamela R. Beilby, Mariángeles Kovacs, Sofía Ibarburu, Valentina Varela, Romina Barreto-Núñez, Samuel C. Bradford, Joseph S. Beckman, Luis Barbeito

**Affiliations:** ^1^Institut Pasteur de Montevideo, Montevideo, Uruguay; ^2^Department of Biochemistry and Biophysics, Oregon State University, Corvallis, OR, United States; ^3^Linus Pauling Institute, Oregon State University, Corvallis, OR, United States

**Keywords:** microglia, ALS, senescence, astrocytes, motor neurons, aging, SASP

## Abstract

Age is a recognized risk factor for amyotrophic lateral sclerosis (ALS), a paralytic disease characterized by progressive loss of motor neurons and neuroinflammation. A hallmark of aging is the accumulation of senescent cells. Yet, the pathogenic role of cellular senescence in ALS remains poorly understood. In rats bearing the ALS-linked SOD1^G93A^ mutation, microgliosis contribute to motor neuron death, and its pharmacologic downregulation results in increased survival. Here, we have explored whether gliosis and motor neuron loss were associated with cellular senescence in the spinal cord during paralysis progression. In the lumbar spinal cord of symptomatic SOD1^G93A^ rats, numerous cells displayed nuclear p16^INK4a^ as well as loss of nuclear Lamin B1 expression, two recognized senescence-associated markers. The number of p16^INK4a^-positive nuclei increased by four-fold while Lamin B1-negative nuclei increased by 1,2-fold, respect to non-transgenic or asymptomatic transgenic rats. p16^INK4a^-positive nuclei and Lamin B1-negative nuclei were typically localized in a subset of hypertrophic Iba1-positive microglia, occasionally exhibiting nuclear giant multinucleated cell aggregates and abnormal nuclear morphology. Next, we analyzed senescence markers in cell cultures of microglia obtained from the spinal cord of symptomatic SOD1^G93A^ rats. Although microglia actively proliferated in cultures, a subset of them developed senescence markers after few days *in vitro* and subsequent passages. Senescent SOD1^G93A^ microglia in culture conditions were characterized by large and flat morphology, senescence-associated beta-Galactosidase (SA-β-Gal) activity as well as positive labeling for p16^INK4a^, p53, matrix metalloproteinase-1 (MMP-1) and nitrotyrosine, suggesting a senescent-associated secretory phenotype (SASP). Remarkably, in the degenerating lumbar spinal cord other cell types, including ChAT-positive motor neurons and GFAP-expressing astrocytes, also displayed nuclear p16^INK4a^ staining. These results suggest that cellular senescence is closely associated with inflammation and motor neuron loss occurring after paralysis onset in SOD1^G93A^ rats. The emergence of senescent cells could mediate key pathogenic mechanisms in ALS.

## Introduction

Amyotrophic lateral sclerosis (ALS) is an adult-onset neurodegenerative disease characterized by progressive upper and lower motor neuron degeneration, leading to muscle weakness and paralysis (Tsai et al., 2017). Although the etiology of ALS remains unknown, age is considered the strongest independent risk factor, most patients being diagnosed between the ages of 50 and 85 (Kiernan et al., 2011). ALS is also characterized by the ineluctable progression of motor deficits, with a variable but short survival of about 20 months (Hardiman et al., 2017). Age of diagnosis is also considered a strong predictor of survival, with hazard ratios progressively increasing each decade for individuals older than 50 years (Crockford et al., 2018). Age-dependence of motor phenotypes has also been described in rodent and fly models (Iguchi et al., 2013; Sreedharan et al., 2015), further supporting ALS as an aging-related condition.

Various studies indicate that motor neuron degeneration in ALS is often associated with increased oxidative and nitrative damage, mitochondrial dysfunction, ER-stress, defective RNA processing, and protein homeostasis (Cassina et al., 2008; Morgan and Orrell, 2016). In parallel, glial cells also become activated, proliferate and display inflammatory features characteristic of gliosis (Philips and Rothstein, 2014; Trias et al., 2018a). These kinds of cellular stresses combined with DNA damage or strong mitogenic signaling in vulnerable cells have the potential to induce cellular senescence (Rodier et al., 2009), a basic and heterogenous mechanism by which damaged cells adapt to maintain survival and prevent potentially deleterious expansion or oncogenic transformation during aging (Munoz-Espin and Serrano, 2014). A fundamental feature of cellular senescence is the arrest of the cell cycle through p16^INK4A^-mediated pathway, which is usually associated with p53 nuclear expression (Prieur et al., 2011). p53 becomes activated in response to a variety of cellular stressors including DNA damage and oxidative stress leading to an increased half-life of the p53 protein, phosphorylation and nuclear translocation. In turn, nuclear p53 can function as a transcription factor to regulate the cell cycle, apoptosis, genomic stability or senescence response (Rufini et al., 2013). Nuclear expression of p16^INK4A^ is considered a robust molecular marker of cellular aging, as its expression increases in a variety of aged tissues (Baker et al., 2011). Another remarkable senescence-associated marker is the loss of nuclear Lamin B1 (Freund et al., 2012), which together with other lamins, is essential to maintain nucleus stability, size and shape (Dechat et al., 2008). The loss of nuclear Lamin B1 in particular is recognized as a senescence marker, functionally associated with the induction of p16^INK4A^ and p53 (Freund et al., 2012).

In addition, senescent cells develop profound phenotypic and functional changes, including an increase in senescence-associated beta-galactosidase (SA-β-Gal) activity, reflecting an increased number of lysosomes (Dimri et al., 1995). In addition, senescent cells enlarge and flatten with a tendency to form multinucleated cell aggregates (Leikam et al., 2015), accumulate oxidative and nitrative damage (Lamoke et al., 2015) and typically display a senescent-associated secretory phenotype (SASP) (Tchkonia et al., 2013), releasing trophic factors, pro-inflammatory signaling molecules, extracellular matrix components and proteases (Rodier et al., 2009). Recent evidence indicate cells expressing senescence markers contribute to the chronic inflammatory environment and progressive degeneration in different tissues from aged animals (Childs et al., 2015), thus acquiring pathogenic significance.

Previous studies in neurodegenerative conditions show that the emergence of glial and neuronal senescent phenotypes displaying inflammatory features contribute to synaptic and neuronal loss (Arendt et al., 1996; Frost, 2016), with the senescence marker p16^INK4a^ being frequently found in a subpopulation of astrocytes (Bhat et al., 2012). In accordance, a senescence phenotype in human astrocytes can be induced by toxic species of amyloid beta in cell cultures (Bhat et al., 2012). Also, brain astrocytes bearing senescence markers have been identified in normal aging and disease conditions (Salminen et al., 2011; Chinta et al., 2013). Both in ALS animal models and patients, aged astrocytes develop senescence markers such as p16^INK4A^, p53, p21, and SA-β-gal, becoming toxic for motor neurons (Martin, 2000; Das and Svendsen, 2015; Turnquist et al., 2016), suggesting a causal pathogenic role in mediating motor neuron loss. To what extent activated microglia follow senescence-associated phenotypes during the course of paralysis progression in ALS remains to be analyzed.

Microgliosis is a recognized pathological feature in ALS patients (Brettschneider et al., 2012). Extensive microglia activation has also been described in transgenic rodent models of inherited ALS carrying SOD1 mutations (Lewis et al., 2014). In SOD1^G93A^ rats, the rapid spread of paralysis is associated with marked microglial cell activation in the surroundings of motor neurons, leading to the emergence of aberrant phenotypes including astrocyte-like hypertrophic cells and giant multicellular clusters (Fendrick et al., 2007; Diaz-Amarilla et al., 2011; Trias et al., 2013). Activated microglia expressing mutant SOD1 in ALS have the potential to induce motor neuron death (Liao et al., 2012; Frakes et al., 2014). Removal of mutant SOD1 transgene from microglia and neurons significantly increases survival in of SOD1^G37R^ mice (Boillee et al., 2006). The unique nature of microglia with the potential for self-renewal and telomere shortening led to the hypothesis that these cells can exhibit senescence (Eitan et al., 2014; Caldeira et al., 2017). Age-dependent and senescence-driven impairments of microglia functions and responses have been suggested to play essential roles during the onset and progression of neurodegenerative diseases (Luo et al., 2010; Spittau, 2017). However, it remains unknown whether deleterious gliosis and phenotypically aberrant glia in ALS are causally associated with the emergence of senescent cells in the degenerating spinal cord.

In this study, we analyzed the expression of senescence markers in the spinal cord and primary cultures of microglia from adult SOD1^G93A^ rats. In an attempt to determine the relationship between the emergence of senescent glia phenotypes and progressive motor neuron loss, we analyzed senescence markers at disease onset and then at advanced paralysis, a time period of only 2 weeks while rapid paralysis develops in SOD1^G93A^ rats.

## Materials and Methods

### Animals and Study Approval

All procedures using laboratory animals were performed in accordance with the international guidelines for the use of live animals and were approved by either the Oregon State University Institutional Animal Care Use Committee or for experiments performed in Uruguay in strict accordance with the requirements of the Institut Pasteur de Montevideo Bioethics Committee under the ethical regulations of the Uruguayan Law N° 18.611 governing animal experimentation. Uruguayan law follows the Guide for the Care and Use of Laboratory Animals of the National Institutes of Health (United States). Male hemizygous NTac:SD-TgN(SOD1^G93A^)L26H rats (Taconic), originally developed by Howland et al. (2002), were bred locally by crossing with wild-type Sprague–Dawley female rats. Male SOD1^G93A^ progenies were used for further breeding to maintain the line. Rats were housed in a centralized animal facility with a 12-h light-dark cycle with *ad libitum* access to food and water. Symptomatic disease onset was determined by a periodic clinical examination for abnormal gait, typically expressed as subtle limping or dragging of one hind limb. Rats were killed well before they reached the end stage of the disease.

### Experimental Conditions

At least three male rats were analyzed for each experiment. Four different conditions were studied as follow: (1) non-transgenic (NonTg) rats of 160–180 days; (2) transgenic SOD1^G93A^ rats of 125–135 days (asymptomatic); (3) transgenic SOD1^G93A^ rats of 170–180 days (onset); and (4) transgenic SOD1^G93A^ rats of 190–200 days (symptomatic 15d paralysis).

### Determination of Disease Onset and End-Stage

As described previously (Trias et al., 2017), all rats were weighed and evaluated for motor activity daily. Disease onset was determined for each animal when pronounced muscle atrophy was accompanied by abnormal gait, typically expressed as subtle limping or dragging of one hind limb. When necessary, end-stage was defined by a lack of righting reflexes or the inability to reach food and water.

### Immunohistochemical Staining of Rat Spinal Cords

Animals were deeply anesthetized and perfused transcardially with 0.9% saline and 4% paraformaldehyde in 0.1 M PBS (pH 7.2–7.4) at a constant flow of 1 mL/min. The fixed spinal cord was removed, post-fixed by immersion for 24 h, and then cut into transverse serial 25 μm sections with a cryostat. Serial sections were collected in PBS for immunohistochemistry. Free-floating sections were permeabilized for 30 min at room temperature with 0.3% Triton X-100 in PBS, passed through washing buffered solutions, blocked with 5% BSA:PBS for 1 h at room temperature, and incubated overnight at 4°C in a solution of 0.3% Triton X-100 and PBS containing the primary antibodies overnight at 4°C. After washing, sections were incubated in 1:1000-diluted secondary antibodies during 3 h at room temperature. Using a stereological approach, p16^INK4a^-positive nuclei, Iba1-/p16^INK4a^-positive cells, Lamin B1/DAPI and ChAT-/p16^INK4a^-positive cells were counted in 25-μm spinal cord sections using confocal microphotograph with a magnification of 25×. At least 15 sections per spinal cord were analyzed (*n* = 3). ImageJ software was used for analysis. For p53 quantification in the spinal cord, p53 density was measured using ImageJ. At least five sections per animal were analyzed (*n* = 3) as previously described (Trias et al., 2018b).

### Antibodies Used

Primary antibodies: 1:200 mouse monoclonal anti-CDKN2A/p16^INK4a^ (abcam, #ab54210), 1:300 rabbit polyclonal anti-p53 (abcam, #ab131442), 1:400 rabbit polyclonal anti-MMP-1 (Novus Biologicals, #NBP1-72209), 1:300 mouse monoclonal anti-Iba1 (Merck, #MABN92), 1:400 muse monoclonal anti-CD68 (abcam, #ab31630), 1:400 rabbit polyclonal anti-ChAT (Merck, #AB143), 1:500 rabbit polyclonal anti-GFAP (Sigma, #G9269), 1:300 mouse monoclonal anti-S100β (Sigma, #S2532), 1:250 rabbit polyclonal anti-Lamin B1 (abcam, #ab16048), 1:300 mouse monoclonal anti-misfolded SOD1 B8H19 (Medimabs, # MM-0070-P), and 1:250 rabbit polyclonal anti-Nitro tyrosine (abcam, #ab42789). Secondary antibodies: 1:500 goat anti-rabbit-AlexaFluor488 or AlexaFluor546 (Thermo Fisher Scientific, #A11035 or #A11034), 1:500 goat anti-mouse-AlexaFluor488, AlexaFluor546 or AlexaFluor633 (Thermo Fisher Scientific, #A11029, #A11030, or #A21052).

### Microglia Cell Culture From Adult Symptomatic SOD1^G93A^ Rats

Microglia cells were isolated from adult symptomatic SOD1^G93A^ rats as previously described with slight modifications (Trias et al., 2013). Rats were terminally anesthetized and the spinal cords were dissected with the meninges carefully removed. The cords were mechanically chopped then enzymatically dissociated in 0.25% trypsin for 10 min at 37°C. Fetal Bovine Serum (FBS) 10% (vol/vol) in Dulbecco’s Modified Eagle Medium (DMEM) was then added to halt trypsin digestion. Repetitive pipetting thoroughly disaggregated the tissue, which was then strained through an 80-μm mesh and spun down. The pellet was re-suspended in culture medium [DMEM + FBS 10% (vol/vol), HEPES buffer (3.6 g/mL), penicillin (100 IU/mL), and streptomycin (100 μg/mL)] and plated in glass-bottom p35 culture dishes for confocal microscopy or 25-cm^2^ tissue culture flasks for flow cytometry analysis. Culture medium was replaced every 48 h.

### Analysis of Aberrant Glial Cells After Phenotypic Transformation

As previously characterized (Trias et al., 2013), primary adult microglia isolated from symptomatic SOD1^G93A^ rats transitioned into aberrant glial cells after 12–15 days in culture. These aberrant glial cells can be maintained in culture for several passages (Diaz-Amarilla et al., 2011). In the present study, passages 2–4 of aberrant glial cells maintained *in vitro* (DMEM-10% FBS) in glass-bottom p35 culture dishes for several days were analyzed for different senescent markers.

### Senescence-Associated-β-Galactosidase (SA-β-Gal) Activity in Cell Cultures

Protocol for β-galactosidase staining was followed as described by manufacturer cell staining kit (Cell Signaling, #9860). Briefly, growth media was removed from the cells and washed with PBS. The 1X fixative solution was added for 15 min at room temperature. After two PBS washes, 1 mL of β-galactosidase staining solution was added overnight at 37°C in a dry incubator. After blue color was developed, β-galactosidase staining solution was removed and plates were mounted using 70% glycerol for long-term storage at 4°C. Both microglia and aberrant glial cells were analyzed at different time points during 12 days. 10×, 20×, and 100× images were acquired using an Olympus CX41 microscope connected to a Evolution^TM^LC Color camera and using ImagePro Express software for acquisition. At least 10 fields per plate were acquired for quantitative analysis using ImageJ software.

### Immunocytochemical Staining of Cultured Cells

Cultured cells were fixed with 4% PFA for 20 min at 4°C and then were washed three times with 10 mM PBS (pH 7.4). Cells were permeabilized using 0.3% Triton-X100 for 20 min. Nonspecific binding was blocked by incubating fixed cells with 5% BSA in PBS for 1 h at room temperature. Corresponding primary antibodies were diluted in blocking solution and incubated 3 h at room temperature. After washing, cells were incubated with secondary antibodies in blocking solution for 1 h at room temperature. For p16^INK4a^ and p53 staining, cells were permeabilized using 2M HCl solution during 15 min at room temperature before incubation with blocking solution. DAPI was used for nuclei staining. At least 10 fields per plate were acquired in a confocal microscope for quantitative analysis using ImageJ software.

### Flow Cytometry of Senescence-Associated-β-Galactosidase (SA-β-gal) Activity

After 12 days *in vitro*, microglia were quantitatively analyzed for SA-β-Gal activity. Briefly, cells were treated with Bafilomycin A1 to inhibit lysosomal acidification, followed by incubation with C_12_FDG (Molecular Probes/Life Technologies), a fluorogenic substrate for β-galactosidase for 2 h at 37°C with 5% CO_2_. Microglia were then rinsed with PBS, harvested by trypsinization, centrifuged, and re-suspended in ice-cold PBS. Cells were immediately run on a Beckman-Coulter FC500 flow cytometer. Data were analyzed using Winlist (Verity Software).

### Flow Cytometry of Cell Cycle Progression

Cells were trypsinized, washed, and centrifuged. The cell pellet was then resuspended in ice-cold 70% ethanol and incubated at -20°C for 30 min for fixation. Subsequently, cells were washed, centrifuged and re-suspended in 0.1% Triton X-100 in Dulbecco’s Phosphate-Buffered Saline (DPBS). RNase A (10 μg/mL) and propidium iodide (20 μg/mL) were added and cells were incubated for 60 min at room temperature. They were then filtered through a 37-μm mesh and run on Beckman-Coulter FC500 flow cytometer and analyzed using Multi-Cycle (Phoenix Software).

### Fluorescence Imaging

Fluorescence imaging was performed with a laser scanning Zeiss LSM 800 confocal microscope with either a 25× (1.2 numerical aperture) objective or 63× (1.3 numerical aperture) oil-immersion objective using Zeiss Zen Black software. Maximum intensity projections of optical sections were created with Zeiss Zen software.

### Statistical Analysis

Quantitative data were expressed as mean ± SEM. Two-tailed Mann–Whitney test or Kruskal–Wallis followed by Dunn’s multiple comparison tests were used for statistical analysis, with *p* < 0.05 considered significant. GraphPad Prism 7.03 software was used for statistical analyses.

## Results

### Expression of Senescence Markers p16^INK4a^ and Lamin B1 in the Spinal Cord of SOD1^G93A^ Rats During Paralysis Progression

**FIGURE 1:**
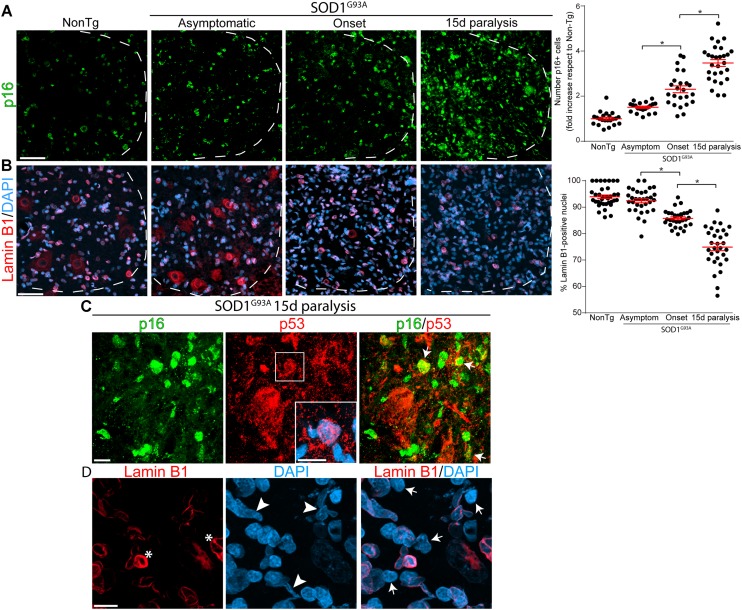
Progressive change in senescence markers p16^INK4a^ and Lamin B1 during paralysis progression in SOD1^G93A^ rats ventral spinal cord. Representative confocal images showing the expression of p16^INK4a^ (green) and Lamin B1 (red) by immunohistochemistry in the degenerating spinal cord of SOD1^G93A^ animals and non-transgenic controls. **(A)** Progressive increase of the senescence marker p16^INK4a^ staining (green) in nuclei from the ventral horn of the spinal cord in symptomatic rat during paralysis progression (white dotted lines indicate the separation of white and gray matter). The graph to the right shows the quantitative analysis of the p16^INK4a^-positive nuclei in the ventral spinal cord. Data are expressed as mean ± SEM; data were analyzed by Kruskal–Wallis followed by Dunn’s multiple comparison tests, *p* < 0.05 was considered statistically significant. Scale bar: 50 μm. **(B)** Confocal microphotographs showing the staining for nuclear Lamin B1 (red) as a marker of non-senescent cells among analyzed groups. The graph to the right shows the quantitative analysis of Lamin B1-positive nuclei in the ventral horn of the spinal cord. Note the progressive loss of nuclear Lamin B1 expression with disease progression. Data are expressed as mean ± SEM; data were analyzed by Kruskal–Wallis followed by Dunn’s multiple comparison tests, *p* < 0.05 was considered statistically significant. Scale bar: 50 μm. **(C)** The confocal images show co-expression of p16^INK4a^ (green) p53 (red) nuclear staining (white arrows) in the ventral horn of degenerating spinal cord at 15d post-paralysis. The inset shows the nuclear localization of p53 in a subset of cells. Scale bars: 10 μm. **(D)** High magnification confocal image showing the loss of nuclear Lamin B1 (red) expression (white arrows) and Lamin B1 invaginations (asterisks) associated to nuclear misshape. Scale bar: 10 μm.

Based on a previous report showing an increase of p16^INK4a^ RNA levels in symptomatic SOD1^G93A^ rats (Das and Svendsen, 2015), we examined the number of p16^INK4a^-positive nuclei and Lamin B1 expression in the ventral horn of the lumbar cord during paralysis progression. Immunohistochemistry analysis revealed a continuous increase in p16^INK4a^ nuclear expression in rats expressing mutant SOD1 as compared with non-transgenic rats (Figure 1A). The number of p16^INK4a^-positive nuclei was significantly increased by 2.3-fold and 3.5-fold at paralysis onset and 15d of paralysis progression, respectively (graph in Figure 1A).

On the other hand, nuclear levels of Lamin B1 significantly declined during paralysis progression in SOD1 rats, 1 out of 4 nuclei exhibiting loss of Lamin B1 at 15d post-paralysis (arrows in Figure 1D), which is significantly different from non-transgenic and asymptomatic SOD1^G93A^ rats (Figure 1B,D). Moreover, the decline in Lamin B1 expression and nuclear Lamin B1 invaginations (asterisk in Figure 1D) were associated with aberrant nuclear shapes (arrowheads in Figure 1D).

Because cellular senescence is characterized by cell cycle arrest through p16^INK4a^- and p53-mediated pathways (Prieur et al., 2011), we also assessed p53 expression in the lumbar ventral horn. As shown in Supplementary Figure S1, p53 immunoreactivity significantly increased in mutant SOD1 rats at paralysis onset and advanced paralysis with frequent colocalization of p16^INK4a^ (Figure 1C). p53 expression levels increased by 1.5- and 2-fold at onset and advanced paralysis, respectively, with respect to age-matched non-transgenic littermates (Graph in Supplementary Figure S1).

### Nuclear p16^INK4a^ and Lamin B1 Expression in Spinal Cord Microglia During Paralysis Progression

**FIGURE 2:**
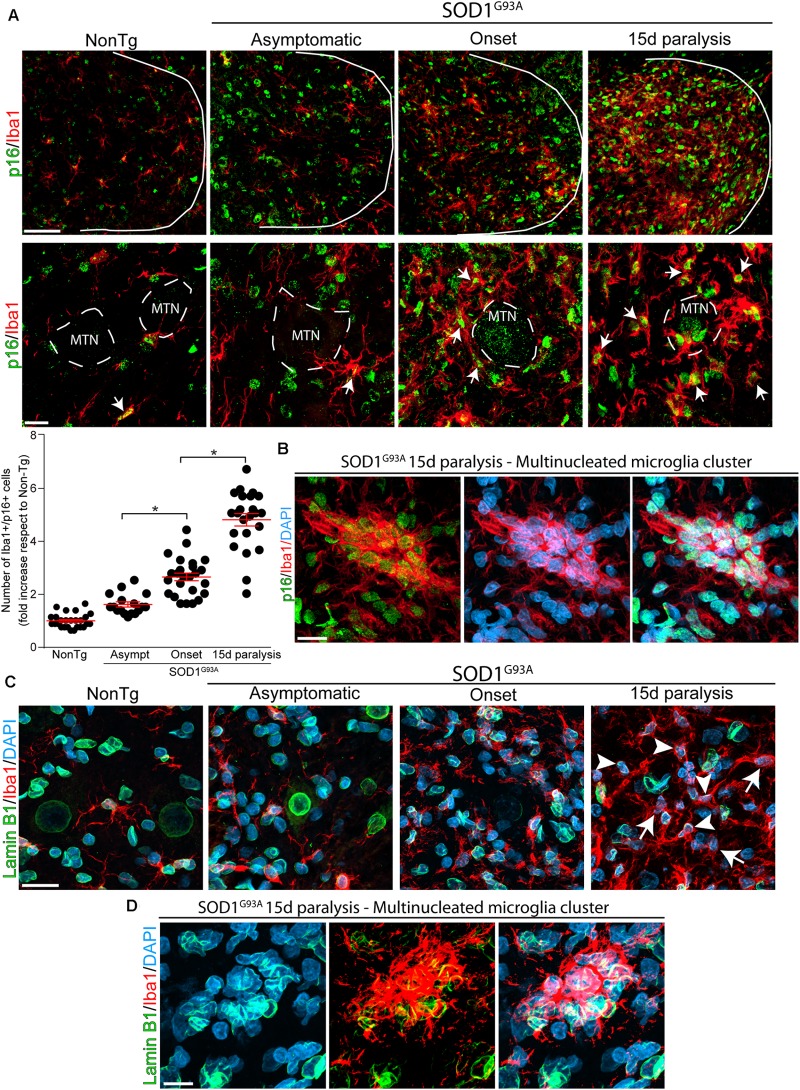
Nuclear p16^INK4a^ and Lamin B1 expression in microglia during paralysis progression. **(A)** Confocal representative images showing the expression of the microglia marker Iba1 (red) and the senescence marker p16^INK4a^ (green) in the non-transgenic, asymptomatic, onset and 15d paralysis SOD1^G93A^ ventral spinal cord. The upper panels (low magnification) show the significant parallel increase of nuclear p16^INK4a^ in Iba1-positive cells during the symptomatic stage of the disease as compared with non-transgenic animals or SOD1^G93A^ asymptomatic stage. Lower panels show at high magnification images of p16^INK4a^-positive swollen microglia (white arrows) surrounding motor neurons (MTN). The graph below shows the quantitative analysis of the expression of p16^INK4a-^ in Iba1-positive cells. Note the sharp increase of p16^INK4a^–positive microglia at 15d post-paralysis. Data are expressed as mean ± SEM; data were analyzed by Kruskal–Wallis followed by Dunn’s multiple comparison tests, *p* < 0.05 was considered statistically significant. Scale bars: 50 μm in low magnification panels and 10 μm in high magnification panels. **(B)** The confocal microphotograph shows a multinucleated microglia cluster expressing Iba1 (red) in a 15d paralysis rat. These Iba1-positive clusters express nuclear p16^INK4a^. Scale bar: 20 μm. **(C)** Representative confocal microphotograph of the ventral spinal cord showing nuclear Lamin B1 expression in Iba1-positive cells (arrowheads). Note the loss of Lamin B1 expression in a subpopulation of cells (arrows) at 15d post-paralysis. Scale bar: 20 μm. **(D)** The confocal microphotograph shows a cluster of multinucleated microglia where Lamin B1 expression is absent in several nuclei (DAPI) at 15d post-paralysis. Scale bar: 20 μm.

Next, we analyzed whether p16^INK4a^ and Lamin B1 were expressed in Iba1-positive microglia, that typically proliferate and become hypertrophic near spinal motor neurons in symptomatic SOD1^G93A^ rats (Trias et al., 2013). As shown in Figure 2A, Iba1-positive microglia express high levels of nuclear p16^INK4a^ (white arrows) in rats developing paralysis. Compared with non-transgenic controls, p16^INK4a^ expression at onset and 15d of paralysis progression significantly increased by 2.6- and 4.8-fold, respectively (graph in Figure 2A). Remarkably, a high density of p16^INK4a^ nuclei was identified in multinucleated microglia clusters (Figure 2B) that are frequently found in the ventral horn of symptomatic SOD1^G93A^ rats (Fendrick et al., 2007), further indicating the correlation of senescence with microglia bearing aberrant phenotypes.

In addition, nuclear expression of Lamin B1 progressively declined in Iba1-positive cells during advance paralysis. Figure 2C shows subpopulation of Iba1-positive microglia that devoid of nuclear Lamin B1 (arrows) coexisting with microglia displaying normal pattern of Lamin B1 staining (arrowheads). Furthermore, nuclear Lamin B1 decline was observed in senescent multinucleated microglia clusters in the lumbar spinal cord (Figure 2D).

Next, we analyzed whether misfolded SOD1 was associated with senescent microglia in SOD1 rats. Misfolded SOD1 is a recognized hallmark of neuronal pathology in ALS linked to SOD1 mutations (REF). As shown in Supplementary Figure S2, misfolded SOD1 was mainly detected in degenerating neuronal somas and dendrites in symptomatic SOD1^G93A^ rats and was not observed in non-transgenic or asymptomatic transgenic rats. However, the presence of misfolded SOD1 in microglia appeared to correspond to neuronal debris being engulfed by phagocytic microglia (arrows in Supplementary Figure S2).

### Nuclear p16^INK4a^ Staining in a Subset of Spinal Motor Neurons and Astrocytes During Advanced Paralysis

**FIGURE 3:**
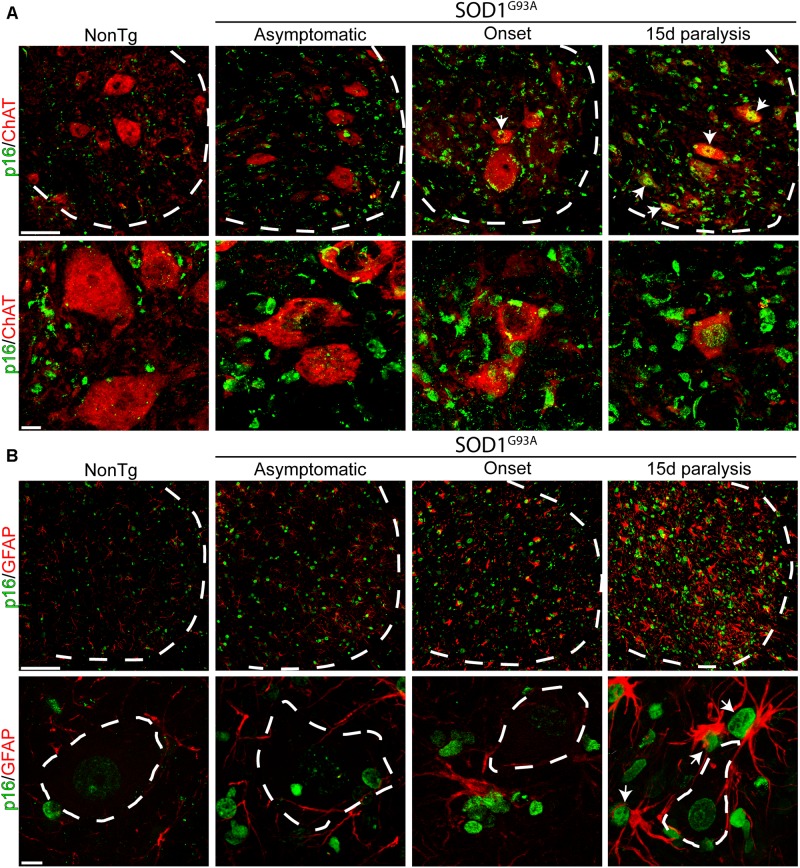
Nuclear p16^INK4a^ expression in a subpopulation of spinal motor neurons and astrocytes. **(A)** Representative confocal microphotograph of the ventral spinal cord of SOD1^G93A^ rats showing ChAT-positive (red) motor neurons at low (upper row) and high (lower row) magnifications. During the symptomatic phase of the disease, a subpopulation of neurons expresses nuclear p16^INK4a^ (white arrows). Dotted white line separate white from gray matter. Scale bars: 50 μm for low magnification panels and 10 μm for high magnification panel. **(B)** Photomicrographs showing p16^INK4a^/GFAP stained lumbar spinal cord sections among groups. Low magnification panels (upper panels) show the notorious increase in the number of p16^INK4a^-/GFAP-positive cells in the symptomatic rats, as compared to low markers co-expression in asymptomatic or non-transgenic rats. Note the expression of p16^INK4a^ marker in a subpopulation of astrocytes that surround motor neurons. Scale bars: 50 μm for low magnification panels and 10 μm for high magnification panel.

Previous reports have shown astrocytes bearing senescent markers in the spinal cord of symptomatic SOD1^G93A^ rats (Das and Svendsen, 2015) as well as in post-mitotic neurons submitted to stress or aging (Jurk et al., 2012). Thus, we looked for p16^INK4a^-expressing astrocytes and motor neurons in the lumbar spinal cord of SOD1^G93A^ rats during onset and 15d of paralysis progression. As shown in Figure 3A, a subset of ChAT-positive motor neurons expressed significant levels of nuclear p16^NK4a^ during the period of rapid motor neuron loss in advanced paralysis. In comparison, motor neurons bearing healthy morphology in asymptomatic SOD1^G93A^ rats were negative to p16^NK4a^, suggesting senescence develops only in damaged motor neurons. Nuclear p16^INK4a^ was also observed in numerous GFAP-positive astrocytes that typically surround motor neurons in the ventral horn of symptomatic SOD1^G93A^ rats (Figure 3B).

### Senescence-Associated β-Galactosidase Activity (SA-β-gal) in Primary Cultures of Microglia From Symptomatic SOD1^G93A^ Rats

**FIGURE 4:**
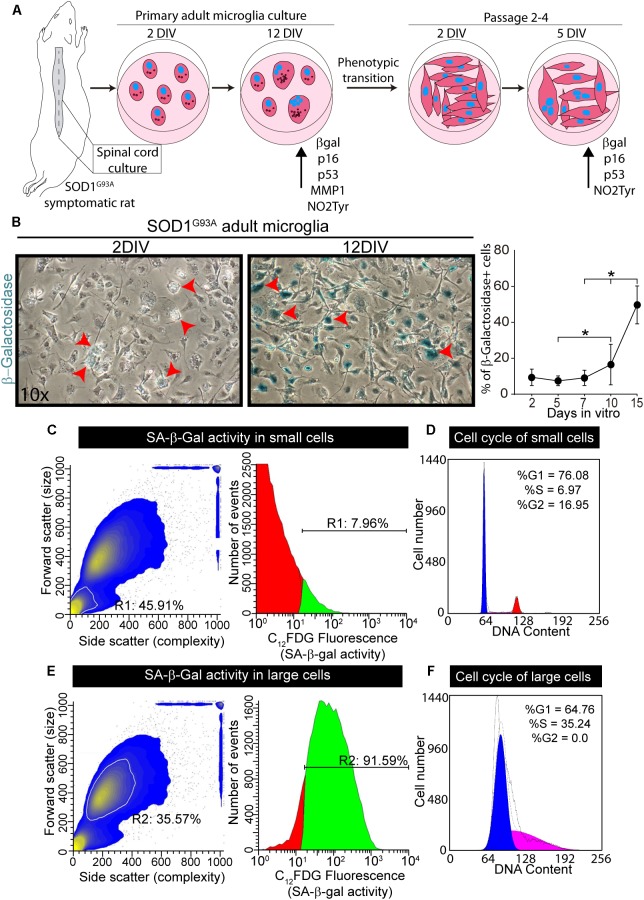
Senescence-associated β-Galactosidase activity in primary cultures of microglia from symptomatic SOD1^G93A^ rats. **(A)** The scheme shows the procedure for adult microglia cell cultures from symptomatic SOD1^G93A^ rats. The spinal cord was plated on p35 culture dishes and SA-b-Gal was measured at different time points. Senescent markers increase their expression after several days in culture. After 2 weeks *in vitro*, microglia transitioned to aberrant glial cells. These transformed cells were also analyzed for SA-β-Gal and senescence markers at different time points in culture. **(B)** The phase contrast microphotographs show SA-β-Gal staining after 2 days *in vitro* (DIV) and 12 DIV. The graph to the right shows the quantitative analysis of SA-β-Gal activity in cultured adult microglia at different time points. Data are expressed as mean ± SEM; data were analyzed by Kruskal–Wallis followed by Dunn’s multiple comparison tests, *p* < 0.05 was considered statistically significant. **(C)** SA-β-Gal activity analyzed by flow cytometry analysis. In the scatter diagram for the smaller population (inside white outline), R1 indicates the percentage of total population encompassed by this subset (45%). The gate for the smaller cell population indicates almost 8% of these cells are senescent. **(D)** The diagram shows the cell cycle analysis for the smaller cell population. **(E)** Scatter diagram for larger cell population (inside the white outline, R2). In the larger cell population, over 90% of the cells demonstrate SA-β-Gal activity. **(F)** The scatter diagram to the right shows the cell cycle analysis for the larger cell population.

We have previously shown that primary spinal cord cultures from symptomatic SOD1^G93A^ rats yield >98% of microglia (Trias et al., 2013). Figure 4A summarizes the behavior of these microglia cultures and its ability to actively proliferate and transform into flat enlarged cells after serial passages. In this context, we explored whether cultured microglia from symptomatic SOD1^G93A^ rats could develop senescence markers as observed in the degenerating spinal cord. Primary cultures of SOD1^G93A^ microglia maintained for 12 days *in vitro* progressively developed positive chromogenic SA-β-gal staining, with ∼8-fold increase between 1 DIV and 12 DIV (Figure 4B). Senescent microglia in cell cultures demonstrated an enlarged, flattened morphology (arrows in Figure 4B), morphological features previously described in other senescent cells (Carnero, 2013).

Flow cytometer analysis of microglia maintained in culture for 12 DIV showed 50% of the cells exhibiting SA-β-gal fluorescent staining (Supplementary Figure S3), with two distinct cell subpopulations, based on size as seen in the scatter diagram of the cells (Supplementary Figure S3). The subpopulation of smaller cells displays only 8% of SA-β-Gal activity and normal cell cycle behavior, corresponding to non-senescence cells (Figure 4C,D). In contrast, 92% of large size cells exhibited SA-β-gal activity and also significant S-phase arrest (Figure 4E,F), the latter being usually associated with inhibition of cell growth, proliferation and senescence in cell cultures (Blagosklonny, 2011).

### Expression of Senescence Markers in Cell Cultures of SOD1^G93A^ Microglia

**FIGURE 5:**
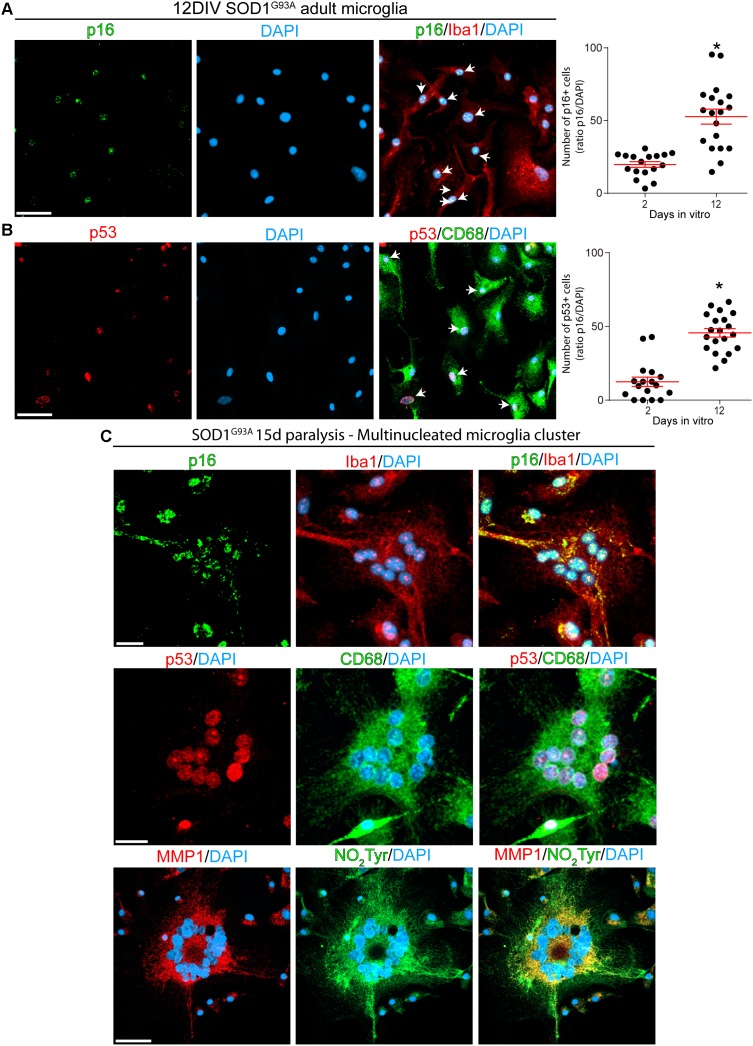
Cultured adult microglia from SOD1^G93A^ symptomatic rats express senescence markers. Immunocytochemistry analysis of senescence markers on microglia isolated from SOD1^G93A^ symptomatic rats at 2 and 12DIV. **(A)** Isolated Iba1-positive microglia express nuclear p16^INK4a^, which expression increase after several days in culture as shown in the graph to the right. Data are expressed as mean ± SEM: data were analyzed by Mann–Whitney test, 2-tailed, *p* < 0.05 was considered statistically significant. Scale bar: 50 μm. **(B)** CD68-positive microglia express increasing levels of nuclear p53 in culture. The graph to the right shows the comparative quantitative analysis of p53 expression. Data are expressed as mean ± SEM: data were analyzed by Mann-Whitney test, 2-tailed, *p* < 0.05 was considered statistically significant. Scale bar: 50 μm. **(C)** After 12 DIV, SOD1^G93A^ isolated microglia form Iba1-/CD68-positive multinucleated giant cells, which express several senescence markers such as p16^INK4a^, p53, and MMP1. Also, these multinucleated cells express high levels of NO_2_Tyr. Scale bars: 20 μm.

Next, we analyzed the phenotypic features of Iba1- and CD68-positive SOD1 microglia at 2- and 12-DIV to identify senescence cellular markers. As shown in Figure 5A,B, approximately 50% of microglia expressed p16^INK4a^ or p53 nuclear staining at 12DIV, as compared with approximately 15% at 2DIV (graphs in Figure 5A,B, and Supplementary Figures S4A,B). 12DIV microglial cells also displayed high levels of MMP-1 and NO_2_Tyr in comparison with 2DIV isolated cells (Supplementary Figure 4C). In addition, p16^INK4a^, p53, MMP-1, and NO_2_Tyr were also found in multinucleated cell aggregates that are frequently found in culture conditions (Figure 5C), reproducing the aberrant features found in the degenerating spinal cord *in vivo*.

The emergence of senescent cells was also observed in serially passaged SOD1^G93A^ microglia cultures, which have undergone a phenotypic transformation (Trias et al., 2013). As shown in Supplementary Figure 5A, the number of SA-β-Gal-positive cells rapidly increased in the following 5 days after plating, ∼50% of these cells also displaying increased p16^INK4a^ and p53 nuclear staining (Supplementary Figures 5B,C).

## Discussion

Amyotrophic lateral sclerosis has been modeled as a multi-step process associating senescence-driven tissue dysfunction with underlying genetic defects and risk factors (Al-Chalabi et al., 2014). In this context, here we report that paralysis progression in a rat model of ALS is characterized by the emergence of numerous microglia, astrocytes and motor neurons displaying phenotypic markers of senescence. Senescent cells seem to be acutely induced after paralysis onset, suggesting a deleterious effect mediated by the ALS neurodegenerative cellular microenvironment and coincident to motor neuron loss. Senescence markers were also observed in cultures of microglia isolated from symptomatic SOD1^G93A^ rats, further indicating the inherent ability of these cells to develop a senescence program with secretory features. In agreement with previous reports showing senescence microglia in aged rodents (Rawji et al., 2016; Theriault and Rivest, 2016), the present data show evidence of a yet unknown mechanism associating microglia activation and cell senescence, with the emergence of secretory phenotypes in a rat model of ALS.

Activation of the p16^INK4a^-pathway is essential for the induction of senescence in a variety of cell types (Prieur et al., 2011). The tumor suppressor p53 also contribute to the induction of cellular senescence in glial cells (Turnquist et al., 2016). We found that the basal levels in p16^INK4a^ and p53 expression were significantly increased in SOD1^G93A^ rats at asymptomatic and paralysis onset stages, respect to age-matched non-transgenic controls. Strikingly, p16^INK4a^ and p53 levels sharply increased after paralysis onset, coincident with extensive spinal cord microgliosis and motor neuron loss occurring in SOD1^G93A^ rats (Howland et al., 2002). Increased levels of p16^INK4a^ and p53 were shown to induce nuclear loss of Lamin B1 (REF). Such a decline in Lamin B1 level constitutes a recognized biomarker of cellular senescence (REF). This is the first report showing a significant increase in nuclear Lamin B1 loss in the degenerating spinal cord of SOD1^G93A^ rats, which was associated with other pathological features of Lamin B1 and nuclear misshape. Senescent microglia showed Lamin B1 loss as well as abnormalities in nuclear Lamin B1 localization pattern. These findings agree with previous reports showing disruption of nuclear Lamin B1 in neural cells associated with Parkinson’s disease and Tau pathologies (Frost et al., 2016; Chinta et al., 2018).

p16^INK4a^ expression and nuclear Lamin B1 decline in microglia were typically observed in cells surrounding the damaged motor neurons. These cells also displayed large size, multinucleated formations as well as MMP-1 and nitrotyrosine staining in culture, suggesting phenotypic aberrations and secretory features. Thus, senescent microglia emerging in the degenerating spinal cord may explain the origin of aberrant glial phenotypes previously described during paralysis progression in SOD1^G93A^ (Diaz-Amarilla et al., 2011; Trias et al., 2013). Taking together, these observations suggest that senescence microglia may result as a consequence of microglia activation, which involves the production of inflammatory mediators and oxidative stress with potential genotoxic activity (Spittau, 2017). Thus, p53 induction in activated microglia from paralytic SOD1^G93A^ rats might not be only related to the senescence program but may also contribute to modulate the inflammatory phenotype as previously described (Aloi et al., 2015).

The finding that microglia isolated from symptomatic SOD1^G93A^ rats develop senescence features in culture conditions further support the inherent ability of these cells to undergo a senescence program. As cultures aged during several days, an increasing number of cells displayed senescence markers such as SA-β-Gal activity, p16^INK4a^, and MMP-1. SA-β-Gal activity, commonly used to distinguish senescent cells (Dimri et al., 1995), is perceptible due to the increased lysosomal content present in senescent cells (Kurz et al., 2000). Interestingly, senescent microglia *in vitro* expressed MMP-1, a marker of SASP (Strzyz, 2016), suggesting this phenotype could define a specific type of microglia polarization in ALS. Levels of matrix metalloproteinases increase with age in many tissues and organs and are associated with the SASP (Freund et al., 2010). MMP-1 levels in glial cells have been shown to be increased in Alzheimer’s disease pathology (Bhat et al., 2012). Recent studies also suggest that metalloproteinases become increasingly dysregulated during disease progression in ALS (Soon et al., 2010), although this has not yet been considered in connection to cell senescence. In addition, senescent microglia isolated from symptomatic SOD1^G93A^ rats showed a tendency to develop cell fusion and multinucleation. This cellular atypia has been previously described in the degenerating spinal cord of SOD1^G93A^ rats (Fendrick et al., 2007). Our finding of microglia bearing SASP is in accordance with our previous reports in microglia in SOD1^G93A^ rats displaying increased transcriptional expression of senescence-associated cytokines and inflammatory factors (Trias et al., 2016), as well as ultrastructural alterations in organelles occurring in cell senescence (Jimenez-Riani et al., 2017).

Cultures containing senescent microglia from symptomatic SOD1^G93A^ rats were characterized by the fact that the emergence of senescent cells was coincident with a robust proliferation capacity of neighboring cells, which could be passaged many serial passages, as previously described (Diaz-Amarilla et al., 2011). Here, we have identified by flow cytometry that senescent microglia exhibited large size and cell cycle arrest, clearly differentiating from a subpopulation of smaller, SA-β-Gal-negative cells, with high proliferative capacity. Thus, SASP microglia in SOD1^G93A^ rats could strongly promote the proliferation of neighboring non-senescent microglia by secretion of soluble factors. In accordance, we have shown that transplantation of SOD1^G93A^ microglia into discrete sites of the lumbar spinal cord on non-transgenic rats, induced a massive microgliosis along the entire spinal cord (Ibarburu et al., 2017).

Because cultured SOD1^G93A^ microglia from the rat paralytic spinal cord shows a high degree of activation, oxidative/nitrative stress and expression of inflammatory genes (Boillee and Cleveland, 2008; Thonhoff et al., 2012), we speculate that the triggering of the senescence program is a consequence of exacerbated cell damage or genotoxic stress, rather than aging *per se*. In accordance, we found that senescent microglia accumulate nitrotyrosine in proteins, indicating oxidative stress producing tyrosyl-radical formation and nitric oxide production (Zhao et al., 2004; Thonhoff et al., 2012). Increased levels of nitrotyrosine residues have been associated with endogenous production of peroxynitrite, a potent cellular oxidant and nitrating agent (Ischiropoulos et al., 1992; Pacher et al., 2007), which has not been previously associated with cellular senescence. In accordance, inflammatory stimulation of macrophages involving increase production of nitric oxide and superoxide also results in p16^INK4a^ expression and SA-β-Gal activity (Hall et al., 2017).

Finally, we found evidence that motor neurons and astrocytes also express nuclear p16^INK4a^ during the symptomatic stage, which might be related to the intriguing accumulation of misfolded SOD1 in motor neuron during advanced paralysis in SOD1^G93A^ rats. This agrees with previous reports showing senescent neurons in aged mice and animal models of Alzheimer’s disease (Jurk et al., 2012; Musi et al., 2018). Because neurons can develop a SASP, they can contribute to induce in inflammation in neighboring cells through the secretion of soluble factors (Appel et al., 2011; Komine and Yamanaka, 2015). Similarly, the finding of senescent astrocytes expressing nuclear p16^INK4a^ in symptomatic SOD1^G93A^ rat spinal cord suggest a role of defective astrocytes in ALS pathology. Astrocytes might exert their neurotoxic effect on motor neurons via the SASP, releasing several proinflammatory cytokines and trophic factors, such as IL-6 (Haidet-Phillips et al., 2011; Das and Svendsen, 2015) and NGF species (Pehar et al., 2004). Astrocytes in ALS rodent models express different senescence markers which potentially turn them into a neurotoxic phenotype for motor neurons both *in vitro* and *in vivo* (Das and Svendsen, 2015; Turnquist et al., 2016). Thus, senescence-associated phenotypes in glial cells and neurons might be relevant pathogenic mechanisms in ALS. It remains unknown, however, whether prevention or eradication of senescence cells in ALS could result in delayed disease progression as has been reported in other neurological diseases (Bussian et al., 2018).

## Conclusion

**FIGURE 6:**
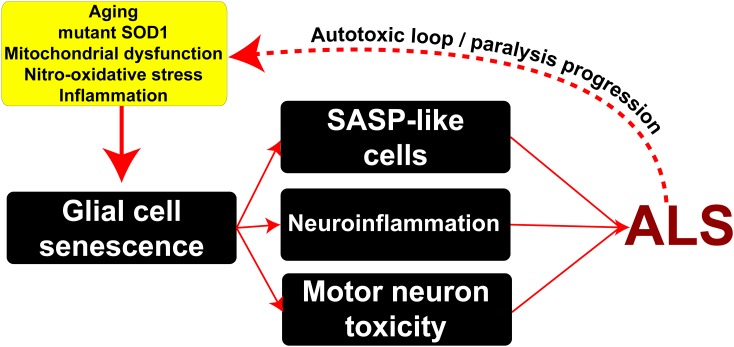
Potential mechanisms underlying the emergence of senescent phenotypes in ALS and pathophysiological consequences. Risk factors such as aging, mitochondrial damage, nitro-oxidative stress, and inflammation may induce the appearance of senescent glial cells in the surroundings of motor neurons bearing SASP. In turn, these cells may exacerbate inflammation and induce motor neuron toxicity through the secretion of soluble toxic factors. This scenario might lead to a pathogenic autotoxic loop promoting the spread of motor neuron pathology and disease progression.

In conclusion, as summarized in Figure 6, here we show for the first time that senescent and secretory microglia emerge during paralysis progression in a rat model of inherited ALS. Risk factors such as aging together with mitochondrial dysfunction and nitro-oxidative damage linked to inflammation likely promote the emergence of senescent glial cells. Subsequently, senescent cells may promote profound changes in the cellular microenvironment through SASPs, exacerbating progressive neuroinflammation and motor neuron toxicity.

## Data Availability

All datasets generated for this study are included in the manuscript and/or the Supplementary Files.

## Author Contributions

ET, PB, LB, and JB designed the research. ET, PB, MK, SI, VV, RB-N, and SB performed the research. ET, PB, SB, LB, and JB analyzed the data. ET, PB, LB, and JB wrote the paper.

## Conflict of Interest Statement

The authors declare that the research was conducted in the absence of any commercial or financial relationships that could be construed as a potential conflict of interest.

## Supplementary Material

Figure S1 Progressive increase in spinal cord p53 expression during paralysis progression Confocal microphotographs show the staining for p53 (red) among analyzed groups. The graph to the right shows the quantitative analysis of p53 intensity. Note the increase in p53 expression with disease progression. Data are expressed as mean ± SEM; data were analyzed by Kruskal–Wallis followed by Dunn’s multiple comparison tests, *p* < 0.05 was considered statistically significant. Scale bars: 50 μm.

Figure S2 Interaction of microglia with degenerating motor neurons expressing misfolded SOD1. Confocal microphotograph showing Iba1-positive microglia clusters (red) surrounding damaged motor neurons accumulating high levels of misfolded SOD1 (green). Arrowheads indicate the microglia/motor neuron clustering. Note that misfolded SOD1 is mainly expressed in neuronal structures during paralysis, while its expression in microglia appears to be associated with the phagocytosis of misfolded SOD1 contained in degenerating neuronal structures (arrows). Scale bar: 20 μm.

Figure S3 Senescence-associated β-Galactosidase activity in primary cultures of microglia from symptomatic SOD1^G93A^ rats. The scatter diagram, a population density heat map, indicates the gate for the sample and includes the entire population of cells. The diagram to the right shows that approximately 50% of the cells demonstrate SA-β-activity.

Figure S4 Expression of senescence markers p16^INK4a^ and MMP1 in cultured adult microglia from SOD1^G93A^ symptomatic rats. Immunocytochemistry analysis of senescence markers on microglia isolated from SOD1^G93A^ symptomatic rats. **(A)** Isolated Iba1-positive microglia after 2 days in culture express nuclear p16^INK4a^ **(A)** and p53 **(B)** in a small subpopulation of cells. Arrows indicate the respective nuclear localization of both markers. Scale bar: 20 μm. **(C)** Progressive increase of MMP1 and NO_2_Tyr in adult cultured microglia. Note the increased expression of MMP1 and NO_2_Tyr between 2 DIV (upper panel) and 12 DIV (lower panel). Scale bar: 20 μm.

Figure S5 Serially passaged SOD1^G93A^ microglia cultures express senescence markers. Senescence marker analysis in phenotypic transitioned SOD1^G93A^ microglia in culture. **(A)** Transitioning microglia population display increasing SA-β-Gal activity (red arrows) at different time points (Passage 4). The graph to the right shows the quantitative analysis of SA-β-Gal activity in transformed microglia. Data are expressed as mean ± SEM; data were analyzed by Kruskal–Wallis followed by Dunn’s multiple comparison tests, *p* < 0.05 was considered statistically significant. **(B)** After several days in culture, transformed microglia express increasing levels of p16^INK4a^ and p53. Also, note the high expression of NO_2_Tyr in those cells that express nuclear p16^INK4a^. Graphs to the right show the quantitative comparative analysis of p16^INK4a^ and p53 at different time points. Data are expressed as mean ± SEM; data were analyzed by Kruskal–Wallis followed by Dunn’s multiple comparison tests, *p* < 0.05 was considered statistically significant. Scale bars: 20 μm.
