# Supplementary figures and images for: Emergence of Microglia Bearing Senescence Markers During Paralysis Progression in a Rat Model of Inherited ALS
Source: Front Aging Neurosci. 2019 Feb 28;11:42. doi: 10.3389/fnagi.2019.00042 (PMC6403180; doi:10.3389/fnagi.2019.00042)

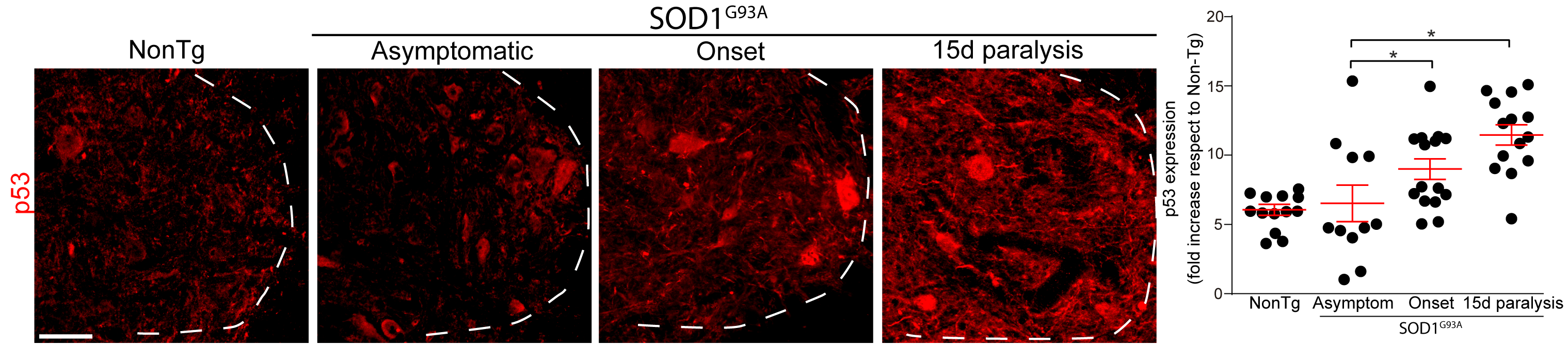

Supplement: Figure S1 — Progressive increase in spinal cord p53 expression during paralysis progression Confocal microphotographs show the staining for p53 (red) among analyzed groups. The graph to the right shows the quantitative analysis of p53 intensity. Note the increase in p53 expression with disease progression. Data are expressed as mean ± SEM; data were analyzed by Kruskal–Wallis followed by Dunn’s multiple comparison tests, p < 0.05 was considered statistically significant. Scale bars: 50 μm. [file Image_1.TIF]

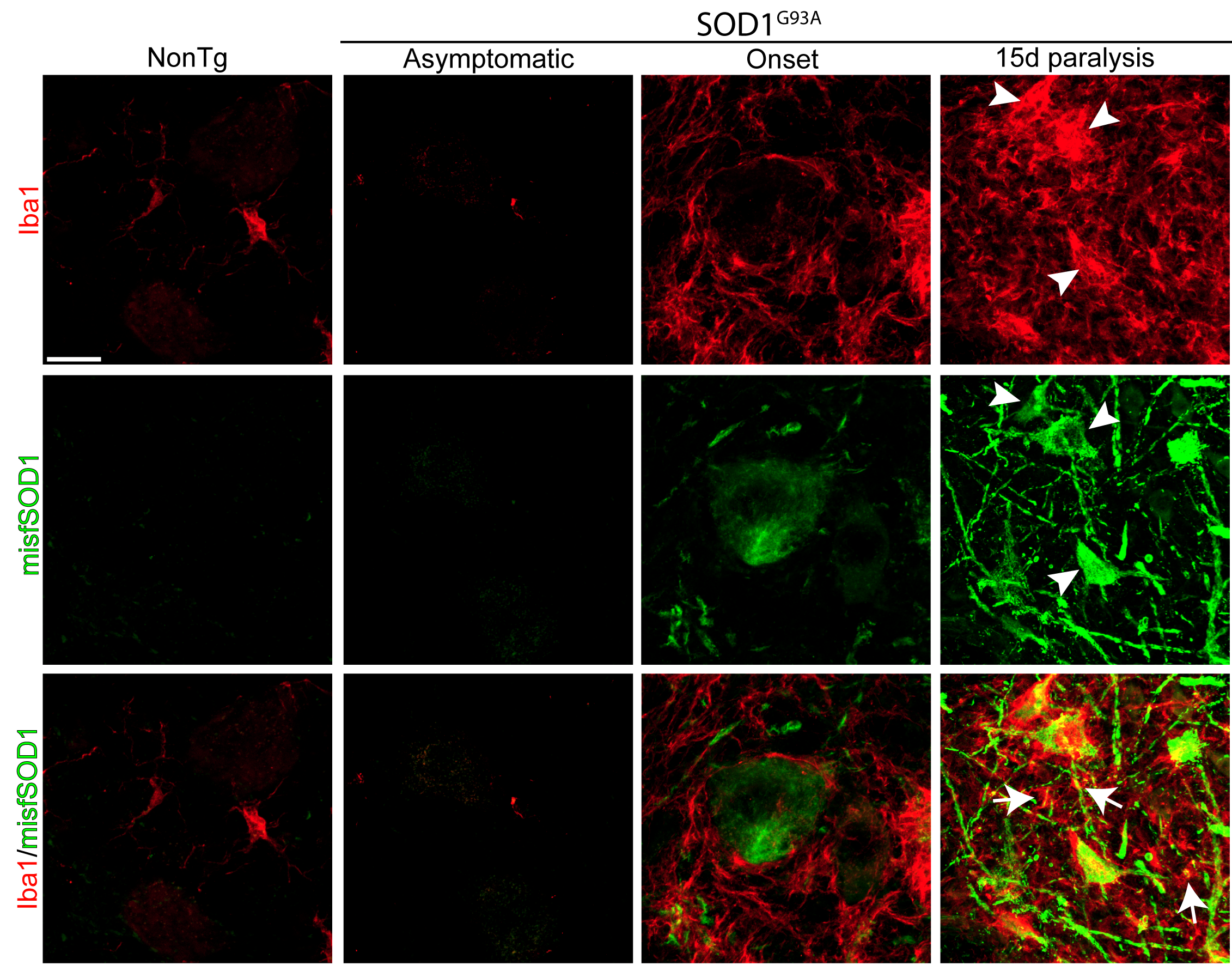

Supplement: Figure S2 — Interaction of microglia with degenerating motor neurons expressing misfolded SOD1. Confocal microphotograph showing Iba1-positive microglia clusters (red) surrounding damaged motor neurons accumulating high levels of misfolded SOD1 (green). Arrowheads indicate the microglia/motor neuron clustering. Note that misfolded SOD1 is mainly expressed in neuronal structures during paralysis, while its expression in microglia appears to be associated with the phagocytosis of misfolded SOD1 contained in degenerating neuronal structures (arrows). Scale bar: 20 μm. [file Image_2.TIF]

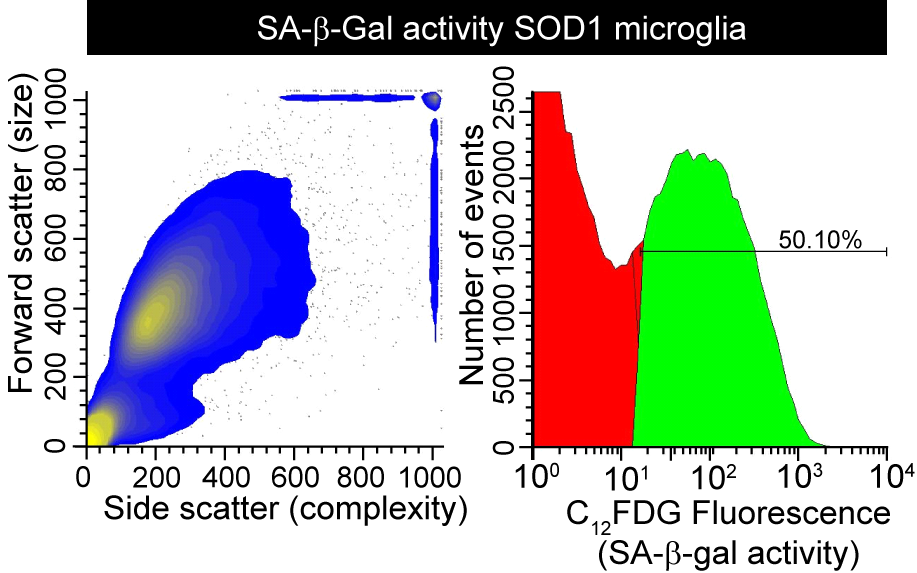

Supplement: Figure S3 — Senescence-associated β-Galactosidase activity in primary cultures of microglia from symptomatic SOD1G93A rats. The scatter diagram, a population density heat map, indicates the gate for the sample and includes the entire population of cells. The diagram to the right shows that approximately 50% of the cells demonstrate SA-β-activity. [file Image_3.TIF]

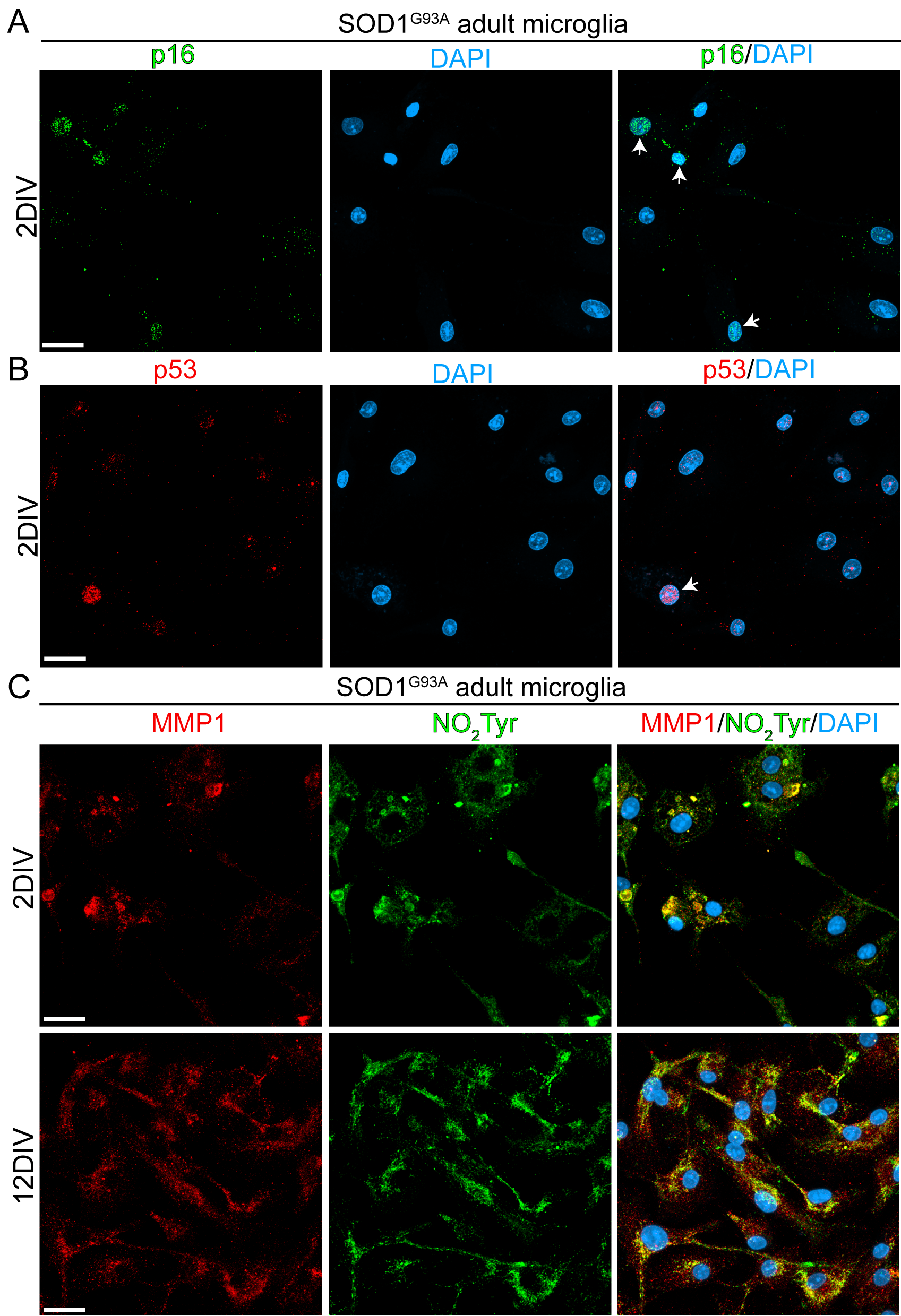

Supplement: Figure S4 — Expression of senescence markers p16INK4a and MMP1 in cultured adult microglia from SOD1G93A symptomatic rats. Immunocytochemistry analysis of senescence markers on microglia isolated from SOD1G93A symptomatic rats. (A) Isolated Iba1-positive microglia after 2 days in culture express nuclear p16INK4a (A) and p53 (B) in a small subpopulation of cells. Arrows indicate the respective nuclear localization of both markers. Scale bar: 20 μm. (C) Progressive increase of MMP1 and NO2Tyr in adult cultured microglia. Note the increased expression of MMP1 and NO2Tyr between 2 DIV (upper panel) and 12 DIV (lower panel). Scale bar: 20 μm. [file Image_4.TIF]

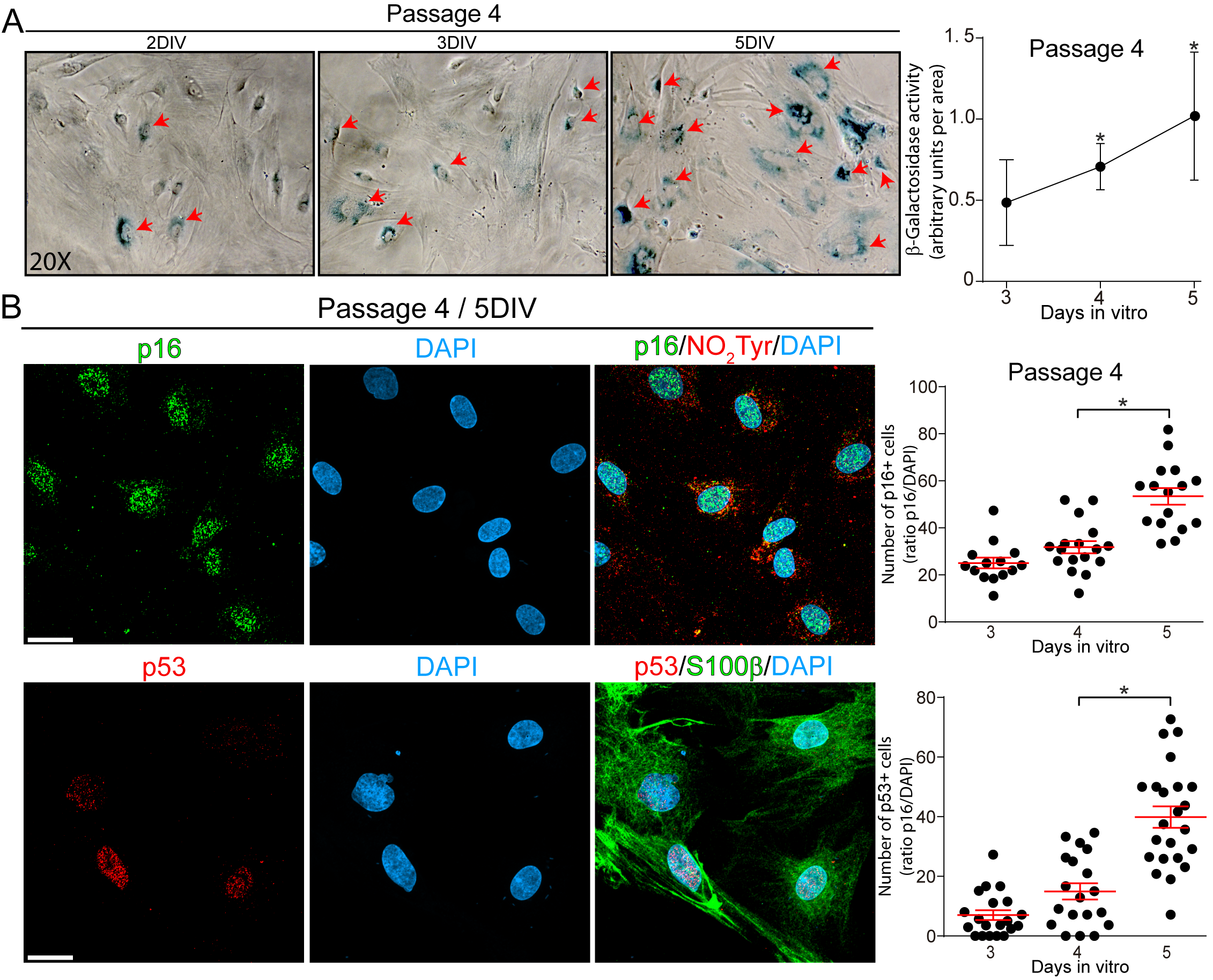

Supplement: Figure S5 — Serially passaged SOD1G93A microglia cultures express senescence markers. Senescence marker analysis in phenotypic transitioned SOD1G93A microglia in culture. (A) Transitioning microglia population display increasing SA-β-Gal activity (red arrows) at different time points (Passage 4). The graph to the right shows the quantitative analysis of SA-β-Gal activity in transformed microglia. Data are expressed as mean ± SEM; data were analyzed by Kruskal–Wallis followed by Dunn’s multiple comparison tests, p < 0.05 was considered statistically significant. (B) After several days in culture, transformed microglia express increasing levels of p16INK4a and p53. Also, note the high expression of NO2Tyr in those cells that express nuclear p16INK4a. Graphs to the right show the quantitative comparative analysis of p16INK4a and p53 at different time points. Data are expressed as mean ± SEM; data were analyzed by Kruskal–Wallis followed by Dunn’s multiple comparison tests, p < 0.05 was considered statistically significant. Scale bars: 20 μm. [file Image_5.TIF]
